# Predictors of Response Rates to a Long Term Follow-Up Mail out Survey

**DOI:** 10.1371/journal.pone.0079179

**Published:** 2013-11-04

**Authors:** Natasha A. Koloski, Michael Jones, Guy Eslick, Nicholas J. Talley

**Affiliations:** 1 Faculty of Health & Medicine, University of Newcastle, Callaghan, Australia; 2 Department of Psychology, Macquarie University, North Ryde, Australia; 3 Department of Surgery, Nepean Hospital, Penrith, Australia; Middlesex University London, United Kingdom

## Abstract

**Objective:**

Very little is known about predictors of response rates to long-term follow-up mail-out surveys, including whether the timing of an incentive affects response rates. We aimed to determine whether the timing of the incentive affects response rates and what baseline demographic and psychological factors predict response rates to a 12 year follow-up survey.

Study design and setting: Participants were 450 randomly selected people from the Penrith population, Australia who had previously participated in a mail-out survey 12 years earlier. By random allocation, 150 people received no incentive, 150 received a lottery ticket inducement with the follow-up survey and 150 received a lottery ticket inducement on the return of a completed survey.

**Results:**

The overall response rate for the study was 63%. There were no significant differences in terms of response rates between the no incentive (58.8%;95%CI 49.8%,67.3%), incentive with survey (65.1%;95%CI 56.2%,73.3%) and promised incentive (65.3%;95%CI 56.1%,73.7%) groups. Independent predictors of responding to the 12 year survey were being older (OR=1.02, 95%CI 1.01,1.05,P=0.001) and being less neurotic as reported on the first survey 12 years earlier (OR=0.92, 95%CI 0.86,0.98, P=0.010).

**Conclusions:**

Psychological factors may play a role in determining who responds to long-term follow-up surveys although timing of incentives does not.

## Introduction

Longitudinal surveys are an extremely important tool in epidemiological medical research as they give clues into the natural history and causality of medical conditions [1,2]. However the outcomes of research based on longitudinal surveys are only valid if an adequate response rate has been achieved and serious non-response biases can be ruled out [3]. Thus attempts to understand what factors may predict higher response rates, in particular the role of incentives in long term follow-up studies is important.

Many of the large scale prospective national longitudinal population-based studies such as the Australian Longitudinal Study on Women’s Health have achieved good response rates over time [4-6]. For example the Australian Longitudinal Study on Women’s Health [4] reported responses rates ranging from 68% and 64% for younger, 90% and 83% for middle aged and 89% and 80% for older cohorts for surveys conducted at 3 and 6 year intervals after the initial mail out [4]. The majority of longitudinal studies like the Australian Longitudinal Study on Women’s Health however have involved repeated contact with participants over relatively regular intervals including mail out of surveys every 3 years, regular newsletters, a project website and telephoning participants to maintain contact details [7]. Clarke et al however demonstrated the feasibility of re-surveying a sample of men who resided in the United Kingdom 25 years after enrollment in the Whitehall study of London Civil Servants [8]. A random sample of 401 study survivors were mailed a request to complete a self administered questionnaire, and then asked to attend their general practice to have their blood pressure, weight, and height measured and a blood sample collected. Accurate addresses were obtained from the health authorities for 96% of the sample. Questionnaires were received from 73% and blood samples from 61% of the sample. Very little is known about potential predictors of response rates in studies that involve recontacting participants after an extended period of time. 

The use of incentives may be one potential factor that could affect response rates in long term follow up surveys. While the evidence for the use of monetary incentives generally favours higher response rates [9-13], not all studies agree [14]. Koloski et al found no significant differences in overall response rates or after reminders when a lottery ticket was used compared to no incentive, but this study was cross sectional [14]. In longitudinal studies the use of incentives is suspected to be favourable although less important in terms of increasing response rates due to fact that the researchers and participants have already formed a research relationship [15]. 

The timing of an incentive, for example whether an incentive is sent with the survey (non contingent) or on the promise of a returned survey (contingent), may also affect response rates [16]. The timing of incentives underlies some key social exchange mechanisms. It is proposed that providing an incentive in advance of participants responding (for example with the initial mail out survey) fosters a trusting relationship between the researcher and participant, which in turn may invoke the participant to reciprocate by responding. In contrast providing a promised incentive contingent upon survey completion suggests to participants that they are being compensated for their time and effort [15,17]. According to Dillman et al, prepayments involve a participant in the research relationship which should in turn increase response rates over a promised incentive [15]. Collins et al (2000) [13] evaluated the timing of a monetary incentive to the eighth wave in a longitudinal study and found that response rates increased with prepayment versus post payment although the effect was very small compared to other cross sectional studies [9]. It is unknown what effect the timing of incentives has in longitudinal studies with minimal contact between surveys. 

It is generally agreed that more females, being older and having a higher educational level is associated with higher response rates [18]. The role of other predictors of response rates to long term surveys after minimal contact is relatively unknown. Pirzada et al (2004) evaluated response rates to a questionnaire 26 years after a baseline examination with minimal interim participant contact [19]. They achieved a response rate of 59.8%. They found female sex, non-White race, older age, lower educational level, cigarette smoking, being overweight, and obesity to be significant predictors of non-response. Their sample however were older, aged 65 years at the 26 year old follow-up and were made up of employees of 84 cooperating Chicago-area organizations. Whether these results would be similar among adults of all ages from the general population is unknown.

We aimed to determine the effect of timing of incentives on response rates. We hypothesised that receiving a lottery ticket with the survey would produce greater response rates compared with a promised incentive on survey completion and no incentive. In addition we aimed to determine what baseline (sociodemographic and psychological) factors predict response rates to a 12 year follow-up survey. 

## Methods

### Participants

This study, approved by The Wentworth Area Health Service Ethics Committee, was conducted as part of a 12 year follow-up population-based study on functional gastrointestinal (GI) disorders in the community. Consent in this study was assumed with the return of a completed questionnaire and this method of consent was approved by the ethics committee. Subjects (n=1775) aged 18 years and older who had participated in a random population survey of 4500 persons 12 years earlier and who agreed in the original survey to be recontacted for future research provided the sampling frame for this study [20]. Four hundred and fifty people were randomly selected from these 1775 people using a random number generator in the SPSS statistical software. The sampling frame for the initial study consisted of the 1996 electoral roll for the local government area of Penrith, which represents 3.6% of the Sydney population. Based on 1996 census data [21], the population of Penrith is representative of the Australian population as a whole, in terms of its sociodemographics and ethnic composition, except that its inhabitants are slightly younger and have a slightly higher socioeconomic status. 

### Measures

#### The Original Survey

The original survey was 32 pages long and comprised a series of questions on functional gastrointestinal symptoms. The questions are initially framed over the past 12 months, but include questions on the presence, frequency, duration and severity of symptoms over the past 3 months (“Did this keep happening for a period of three months or more?”), which enabled a diagnosis of 19 functional gastrointestinal disorders to be made based on Rome slightly modified Rome II criteria [22] as well as the presence of any functional gastrointestinal disorder which was used in these analyses. This questionnaire has been repeatedly tested and very carefully validated [23] with little missing data observed for the individual questions (1.2% to 3%) [20]. 

Also, included in the original survey were psychological measures. These consisted of the Delusion Symptom States Inventory (DSSI) [24]. This scale contains 7 items for anxiety and 7 items for depression and was originally chosen for the 1997 survey because it has published clinical cut-offs and is a strong clinical measure that is well validated [24]. The Eysenck Personality Questionnaire (EPQ) [25] contains 24 items and was used to assess premorbid neuroticism and extroversion. Also included was the Sphere [26], which provided a score for somatic distress over the past 4 weeks. This score closely corresponds to the DSM-III-R diagnosis of somatization. This disorder is characterized by a combination of pain, GI, sexual, and pseudoneurological symptoms. The SF-12 [27], a generic quality of life measure assessing mental and physical functioning over the past 4 weeks, was also included.

Demographics in this questionnaire included gender, age, educational level (completed high school level education or above versus below high school level education) and country of birth (Australian born versus not).

#### The Follow-up Survey

While minor changes were made to the follow-up survey to ensure that FGIDs can also be diagnosed using more recent versions of the Rome criteria, all the Rome II questions from the baseline survey were also included [22]. The questionnaire also contained the Delusion-Symptom-States-Inventory [24] and basic demographic questions for cross-checking purposes. 

### Procedure

The Australian Electoral Commission provided us with up-to-date addresses for participants at the beginning of the study. The sample was then randomly divided into 3 experimental groups according to a random block procedure. One group was randomised to receive an inducement with the survey, another group received a promised inducement on return of a completed survey and the third group received no inducement. Each group consisted of 150 subjects, respectively. 

The follow-up protocol for non-responders was based in part on Dillman’s Total Design method [15] and consisted of a reminder/thank-you letter sent to all participants one week after the initial survey, a replacement survey sent to non-responders only at week 4 as well as a thank-you/reminder letter sent to these non-responders at week 5. All participants were informed in the information sheet that they may indicate refusal to take part in the study or stop reminder letters at any time either verbally over the telephone or by email or by sending back an uncompleted survey.

The cover letter included several elements designed to increase the subject’s personal interest in the study. These included a personal salutation, a scanned version of the investigator’s handwritten signature, an explanation of the nature and importance of the research, and reassurance of confidentiality. The covering letter was the same for all three groups, with the exception that the letter sent to subjects receiving an incentive with a survey that stated “In appreciation for the time taken to help with this important survey we have included a $1 instant ‘scratchie’ lottery ticket. Good luck”. The second group who were promised an inducement on return of the completed survey, the cover letter included the following statement “In appreciation for the time taken to help with this important survey we will send you a $1 instant “scratchie” lottery ticket once we receive your completed survey. Good luck!” This inducement had the potential to win $50,000. Additional measures to maximise response rates included a clear affiliation with the University of Sydney and Nepean Hospital, an easy to understand attractive coloured questionnaire booklet with probes to skip to the next section if questions do not apply, postage stamps on envelopes and inclusion of a reply-paid envelope. 

### Statistical Analyses

Response rates were calculated and reported with 95% exact confidence intervals to provide a sense of the degree of uncertainty around the sample estimate. The responder rates were compared across randomised incentive groups using the Pearson Chi-Square test. Although randomisation minimizes the risk of confounding or suppressor effects the incentive group comparisons were repeated controlling for demographics (age and gender) and baseline psychological state (anxiety, depression, neuroticism and extraversion) via unconditional logistic regression which modelled the probability of being a responder. 

Univariate contrasts of responders and non-responders were undertaken using Mann-Whitney tests for numeric characteristics, such as age, and the Pearson Chi-Square test for categorical characteristics such as incentive group. Multivariable models of predictors of response from baseline variables were conducted using unconditional logistic regression which modelled the probability of being a responder. Hence odds ratios >1.0 indicate that variable is associated with higher probability of response whereas odds ratios <1.0 indicates an association with lower probability of response. Due to the non-orthogonal design of the study it was considered relevant to undertake a model reduction strategy to identify those predictors of response probability that were statistically independent. This was undertaken using backward elimination since this approach takes into account the full correlation structure among the potential predictors. The final multivariate model included just those predictors with statistically significant, independent discrimination of responders from non-responders. A two-tailed p-value <0.05 was used to retain potential predictors. An insight into how well statistically significant predictors of response probability differentiate responders from non-responders comes from the area under the Receiver-Operator-Characteristic curve. An area of 0.5 is equivalent to the toss of a coin whereas an area of 1.0 indicates perfect discrimination.

### Data availability

The data reported in this manuscript was collected under an ethics clearance that requires data confidentiality. If a reader would like specific data summaries we will endeavour to provide this on request. 

## Results

### Response rate

The overall response rate to the survey was 63.3% (95%CI 58.3%-68.2%) (n = 240). The sample size was reduced by 69 ineligibles (n = 50 return to senders and n= 19 deaths). We also looked at the response rate after the first mail out and week 1 reminder letter and found that 197 out of 381 returned the survey, giving a response rate of 52.0% (95%CI 46.8%-57.1%). Thus, sending the Week 4 replacement survey and week 5 reminder/thank you letter resulted in another 11% of surveys being returned.

### Effect of lottery ticket inducement timing

The final response rates for the three lottery ticket incentive groups was n=77 (58.8%; 95%CI 49.8%,67.3%) for no incentive, n=84 (65.1%; 95%CI 56.2%,73.3%) when a lottery ticket was sent with the survey and n=79 (65.3%; 95%CI 56.1%,73.7%) when a lottery ticket was promised on the return of a completed survey. There were no significant differences in terms of response rates between the three lottery incentive groups (P=0.47). 

Although the lottery groups were randomly allocated the possibility of suppressor effects was considered by retesting difference in response rates between incentive groups after controlling for age and gender (p=0.3) and controlling for age, gender and baseline anxiety, depression, extraversion and neuroticism (p=0.6) indicating that candidate suppressing variables were not the cause of the lack of differences in response rates.

 After the initial mailout and week1 reminder letter the response rates for the three lottery ticket conditions were n= 64 (48.9%;95%CI40.0%,57.7%) for no incentive, n= 73 (56.6%;95%CI47.6%,65.3%) for a lottery ticket with the survey and n=60 (49.6%;95%CI 40.4%,58.8%) with a promised lottery ticket inducement. Again, there were no significant differences between the lottery incentive groups on response rates after the first mail out (P=0.39).

### Baseline predictors of response rates to the 12 year follow-up survey

Univariately we found that responders to the 12 yr follow-up survey were significantly older than non-responders (Table 1). Responders to the 12 yr survey were significantly less anxious, neurotic and somatically distressed compared with non-responders (Table 1). Responders to the survey also had a significantly better quality of life in terms of mental functioning than non responders, although no difference in terms of physical functioning was observed between responders and non-responders (Table 1). Responders and non- responders were not significantly different in terms of gender, education, nationality, depression or extroversion (Table 1). The number of people with a functional gastrointestinal disorder at baseline was also similar between responders and non-responders (Table 1).

**Table 1:** Characteristics of responders and non-responders to a 12 year follow up survey.

**Variable**	**Responders**	**Non responders**	**P value**
Female n (%)	126 (52.5)	79 (56.8)	0.41
High School education or above n (%)	142 (59.2)	80 (57.5)	0.70
Australian born n (%)	181 (75.4)	98 (70.5)	0.15
Age Mean (std dev)	45.1 (12.9)	38.9 (15.3)	0.0001
Anxiety Mean (std dev)	2.8 (2.9)	3.7 (3.6)	0.03
Depression Mean (std dev)	1.8 (2.7)	2.4 (3.5)	0.09
Neuroticism Mean (std dev)	4.0 (3.3)	5.1 (3.4)	0.002
Extroversion Mean (Std dev)	5.7 (3.0)	6.1 (3.0)	0.19
Somatic distress Mean (std dev)	43.1 (56.1)	49.1 (50.7)	0.002
Mental Functioning Mean (std dev)	46.6 (8.7)	44.0 (9.7)	0.007
Physical Functioning Mean (std dev)	50.8 (7.4)	50.5 (8.0 )	0.78
Functional Gastrointestinal Disorder Diagnosis n (%)	96 (40.0)	63 (45.3)	0.31

### Multivariate Analyses

In a multivariate model that considered gender, age, functional gastrointestinal disorder diagnosis, anxiety, depression, neuroticism, extroversion, mental and physical functioning and somatic distress, we found being older (OR=1.02, 95%CI 1.01,1.05, P=0.001) and less neurotic at baseline (OR=0.92, 95%CI 0.86,0.98, P=0.01) to be independent predictors of responding to a 12 year follow-up survey. After controlling for these two predictors of response probability none of the remaining variables contributed incrementally to differentiating responders from non-responders. The logistic model incorporating these two variables is associated with an AUC of 0.66 indicating discrimination better than a coin toss but also clearly imperfect.

## Discussion

This study has provided important information on the timing of an incentive and other predictors of response rates to a long-term follow-up survey with minimal contact since the original survey. Very few studies have investigated predictors of response rates to long term follow-up surveys with minimal interim contact despite the fact that these types of studies are becoming more feasible with new technology available for identifying participants up to date addresses. Longitudinal studies can offer valuable insights into disease pathology but achieving satisfactory response rates are required. 

The results from the current study can reassure researchers that good response rates can be achieved in long-term follow-up studies despite maintaining minimal contact with participants. We achieved a very good response rate of 63% in this study which although is lower than achieved by some large scale longitudinal studies that have maintained regular contact with participants [4-6], was in line with response rates associated with younger cohorts enrolled in longitudinal studies [4]. At least half of our initial sample was aged 45 years and lower at baseline. Our response rate was comparable with that reported by Pirzada et al (2004) who obtained a 59% response rate to a mail out survey after 26 years of minimal contact [19]. This is despite the fact that Pirzada et al did not use reminder letters and there was no contact during the 27 years of follow-up. While the majority of this sample had not received any contact since the original survey, there was a small proportion (8%) who had participated in some smaller sub studies in the years following the initial 1997 survey but all participants had not been contacted by the researchers since 2001, a time frame of at least 8 years. We also followed the well established Dillman’s Total Design Method [15] for the follow up of non-responders. Without our replacement survey, our response was 52%. Thus including the replacement survey as well as a week 5 reminder letter resulted in the return of another 11% of surveys, which brought our overall response rate into the more acceptable range for publication [28]. 

While incentives are commonly used as a strategy for increasing response rates in mail surveys, the majority of studies generally favour non contingent over contingent incentives [16]. For example Gneezy and Rey-Biel (2012) et al in a cross sectional study found that compared to no payment, very small contingent payments lowered the response rate to a mail out survey while small non-contingent payments raised the response rate [16]. They support the contention that small unconditional gifts are effective in triggering reciprocity [29]. However we did not find any significant differences in terms of response rates between sending out an incentive with the survey, a promised incentive or no incentive. This extends our previous cross sectional work with the same cohort where the use of an instant scratchie lottery incentive did not significantly increase response rates over no incentive. Thus, it could be that the use of other strategies including a personalised cover letter, attractive easy to understand coloured survey, postage stamps and affiliations with the local university and hospital were more important in increasing response rates in these studies. 

It is also possible that the mechanisms linked to timing of incentives are different for recruiting participants into a study for the first time compared with retaining them in a longitudinal study [15,17]. For example according to social exchange theory, offering a prepayment to a participant in the hope of creating a trusting research relationship will not be as effective in long term studies where this relationship has already been established previously [15,17]. It is important to note however that despite our lack of a significant difference in regards to the timing of an incentive and response rates we did report 5% more surveys being returned when the incentive was sent out with the survey. The decision for researchers is whether a few extra surveys are worth the cost involved with using incentives. In times where response rates to mail surveys are on the decline [30,31], particularly in longitudinal surveys, these extra surveys may make the difference between a study being publishable or a study having a larger enough sample size to work with.

Similar to previous studies [19] we did find found that being older was a significant predictor of responding to the follow-up survey. This may be due to the fact that older people are considered to be less geographically mobile than younger people and more readily contactable for follow up mail out surveys. We did not find other demographic factors that have been found to be associated with higher response rates in other longitudinal studies [19] including being female and higher educational level to be significantly different between responders and non-responders in the current study. The reasons for this discrepancy may be that those who had originally participated in the 1997 survey were slightly more likely to be female anyway and may have been more highly educated to begin with, although this latter possibility cannot be accurately determined as such data along with other demographic factors are not available for non-responders to the original survey. The survey however was carefully designed to be easy to complete with a 6^th^ grade reading age level so it is possible that educational level may not have been a major influence in the decision to participate in this study.

 Of interest we did observe that psychological factors appeared to play a role in determining who will participate in long-term follow-up surveys. We found that people at baseline who were less neurotic were independently more likely to respond to the follow-up survey. Neuroticism is a personality trait that is characterised by persistently enduring negative emotions and over interpreting situations as threats [32] and this may be responsible for not responding to a mail out survey. Non-responders to the 12 year follow up survey also reported higher levels of anxiety, somatic distress and had poorer mental functioning on the 1997 survey although these were not independent predictors of response rates. While the relationship of psychological state and response rates in long term follow up studies is lacking, others have also confirmed that study nonparticipants have higher disease and mortality rates, poorer health status, and lower levels of functioning than study participants [33,34] and this may be particularly the case in follow-up studies such as this where participants could potentially have had these symptoms for a long period of time. These results suggest that responders to long-term surveys may be psychologically healthier than non-responders and this may be important to consider as a potential bias in studies where psychological distress is an outcome. We did not find having a functional gastrointestinal disorder at baseline to be significantly associated with response rates to the 12 year follow-up survey.

This study had several strengths. We undertook a population-based survey of 450 people from the general community. While the majority of current addresses were able to be used, the provision of a return address on the envelopes meant we should have captured the majority of return to senders and were then able to take them out of the total denominator. A small percentage (8%) had participated in earlier sub studies which may have affected the research relationship but this may have been beneficial or detrimental (participants feeling burdened by being asked to participate again). All participants however had not received any contact from the research team for the last 8 years. The results of this study however cannot be generalised to other types of incentives. While the use of an instant ‘scratchie’ lottery ticket has been shown to be effective in increasing response rates, improving the representativeness of the sample and reducing item nonresponse [35] others have shown that monetary incentives especially cash are more effective than non-monetary incentives [36,37] and should be investigated in future studies.

In summary, psychological factors may play a role in determining who responds to long term follow-up surveys although timing of incentives does not.
